# Coping With COVID-19: Mindfulness-Based Approaches for Mitigating Mental Health Crisis

**DOI:** 10.3389/fpsyt.2021.563417

**Published:** 2021-03-23

**Authors:** Elena Antonova, Karoly Schlosser, Rakesh Pandey, Veena Kumari

**Affiliations:** ^1^Divison of Psychology, Department of Life Sciences, College of Health, Medicine and Life Sciences, Brunel University London, London, United Kingdom; ^2^Centre for Cognitive Neuroscience, College of Health, Medicine and Life Sciences, Brunel University London, London, United Kingdom; ^3^Department of Psychology, Goldsmith, University of London, London, United Kingdom; ^4^Department of Psychology, Faculty of Social Sciences, Banaras Hindu University, Varanasi, India

**Keywords:** COVID-19, mental health, mindfulness, depression, anxiety, PTSD, psychosis, coping

## Abstract

The novel coronavirus disease COVID-19 that first emerged in Wuhan, China, in Nov-Dec 2019 has already impacted a significant proportion of the world population. Governments of many countries imposed quarantines and social distancing measures in 2020, many of which remain in place, to mitigate the spread of the SARS-Cov-2 virus causing the COVID-19 disease. The direct impact of COVID-19 on people infected with the virus, their families and the health care workers, as well as the impact of the mitigation measures such as quarantine, social distancing, and self-isolation on the rest of the population have contributed to a global mental health pandemic, including anxiety, depression, panic attacks, posttraumatic stress symptoms, psychosis, addiction, obsessive-compulsive disorder, and suicidality. These effects are present acutely (for example, due to fear of contamination or losing loved ones, effects of quarantine/isolation, withdrawal of community and social services, etc.) and may continue long after the pandemic is over (for example, due to bereavement, unemployment, financial losses, etc). The COVID-19 pandemic has triggered mental health problems in people without previous history of mental illness, as well as worsened the symptoms in those with pre-existing psychiatric diagnosis. Therefore, the global effort is called for to deal with this mental health pandemic secondary to COVID-19 itself to address the emergence of new as well as the exacerbation of the existing mental health issues. Conversely, this global context provides an extraordinary opportunity for studying individual differences in response to and resilience in the face of physical and psychological threat, challenge to “normal” way of life, and long-term uncertainty. In this viewpoint article we outline the particular suitability of mindfulness, its skills and mechanisms, as an approach to the prevention and management of mental health issues, as well as to the promotion of well-being and building the foundations of adaptability and flexibility in dealing with the long-term uncertainty and profound changes to the social, economic, and possibly political systems as this pandemic continues to unfold.

## Introduction

The novel coronavirus disease COVID-19 that first emerged in Wuhan, China, in Nov-Dec 2019 is continuing to spread rapidly with over 94 million confirmed cases world-wide (1). To minimize its human-to-human transmission rates, governments of many countries imposed quarantines and social (physical) distancing measures in the first quarter of 2020 that lasted for months, and have been reinstated in the last quarter of 2020 in many countries, including the UK, that are witnessing a severe outbreak due to more transmissible variants of the SARS-Cov-2 virus. The direct impact of COVID-19 on people infected with the virus, their families and the health care workers, as well as the indirect impact of the mitigation measures such as quarantine, social distancing and self-isolation on the rest of the population has led to a global mental health crisis (2–5) that calls for a global effort in dealing with. This global context also provides an unprecedented opportunity for studying the factors and mechanisms underlying individual differences in response to, and resilience in the face of, an unprecedented challenge to one's “normal” way of life in the context of physical as well as psychological threat and uncertainty.

The international scientific community has risen to the challenge, first in the fields of virology and epidemiology, with the psychology and neuroscience of mental health gathering a strong momentum in appraising the evidence from previous pandemics as well as the data generation during the current pandemic to inform governmental policies for public health interventions and provision. A rapid evaluation of evidence by Brooks and colleagues (6) published in Lancet shortly before the start of quarantine period in the UK and many other countries reviewed the psychological impact of quarantine during previous pandemics and found most studies to report negative psychological effects including post-traumatic stress symptoms, confusion, and anger. The identified stressors for worse mental health outcomes included longer quarantine duration, infection fears, inadequate supplies, inadequate information, financial loss, frustration, boredom, and stigma, with suggestions of long-lasting effects for the mental health issues. It is becoming increasingly evident that the same stressors and psychological effects, as well as information transparency, supplies of necessities, and appeals to the altruistic behavior for the wider societal benefit as mitigators are indeed relevant to the current pandemic (7).

There is significant evidence that the COVID-19 pandemic has triggered mental health problems in people without any previous history of mental illness and worsened the symptoms in those with pre-existing psychiatric diagnosis (3–5). The common mental health problems reported during the Wuhan lockdown imposed between January and March of 2020 included fear, anxiety, depression, and sleep problems in patients with COVID-19 infections, close contacts, the public, and the health care professionals (8–10). The mental health situation in Wuhan was particularly challenging for the subpopulations with existing mental health difficulties, with patients having serious psychiatric needs being considered to be highly vulnerable population to contract COVID-19 (11). Hundreds of patients with psychiatric disorders, as well as mental health professionals, were infected in China (12). Similar findings have since then emerged from other countries and cultures (4, 5). There are also reports of coronavirus infection-related delusions and hallucinations in vulnerable people from China (13) and elsewhere (14). A range of negative mental health consequences are likely during the pandemic (for example, due to fear of catching coronavirus infection, underlying health conditions, losing loved ones due to COVID-19, withdrawal of other healthcare and community services, or a consequence of quarantine measures) and for years to come after it is over (for example, trauma due to the experience of illness or bereavement, survival guilt, unemployment and financial losses) (6, 15).

Quarantine is a necessary preventive measure during major infectious outbreaks but its negative mental health impacts, especially if lasting for more than a few weeks, are also well-documented (6). According to the poll published by the Office of National Statistics (16) in the UK, 85.2% respondents are worried about the effect that coronavirus is having on their life, with 53.1% having stated the coronavirus pandemic has impacted their well-being, and 46.9% reporting high levels of anxiety. Importantly, the general population survey by Ipsos MORI (17) revealed that becoming unwell with COVID-19 disease was ranked lower than the concerns regarding psychological effects of social (physical) distancing on well-being, including increased anxiety, depression, stress, and other negative feelings. The survey by the mental health charity MQ highlighted concerns about the impact of social isolation and increased feelings of anxiety and depression in people with lived experience of a mental health issue. In addition to growing concerns about the impact of isolation on mental health in the general public, there is a clear recognition that the coronavirus pandemic will put the healthcare workers at risk of burn-out and post-traumatic stress disorder. Even after the quarantine/lockdown measures are eased in the UK and elsewhere, we will have to live most likely with a new “normal” for an extended period of time, facing social and economic uncertainty, with a high fear/probability of future waves of infection spread, followed by the periods of stricter restrictions on our way of life to contain and manage the pandemic. The short and long-term impacts on the global economy is likely to have a devastating effect on mental health, affecting ever increasing number of people worldwide.

In what follows we provide a theoretical perspective on why mindfulness-based approaches might be well-suited for responding to the current mental health challenges and managing the short- as well as long-term impact on mental health of the pandemic itself and measures to mitigate it. It should be noted that by “suitability” we do not imply “superiority” to other alternative or complimentary approaches that might also be suitable in the context of COVID-19 pandemic (18, 19). We use the term “approaches” (which is broader than “interventions”) to discuss the suitability of mindfulness and its skills that could be trained by many different means, including in the context of mindfulness-based interventions, using online apps/mp3s, joining online drop-in sessions led by experienced mindfulness instructors/teachers, etc. The systematic reviews and meta-analysis of mindfulness-based interventions (MBIs) on a range of conditions have been conducted (20, 21), including the effects on depression, anxiety and stress reduction in older adults (22) and post-traumatic stress (23). We acknowledge that more rigorous studies are needed to evaluate the efficacy of MBIs and further clarify their mechanisms of action [for the evaluation of the state of the field in terms of methodological rigor please see (24, 25)]. Here we aim to present an appraisal of mindfulness's suitability in terms of its theoretical underpinnings and known mechanisms of action in the context of the stressors, demands, and challenges of COVID-19 pandemic.

## Mindfulness Skills, Transdiagnostic Mechanisms, and Relevance to the COVID-19 Pandemic

### What Is Mindfulness?

*Mindfulness*, one of the words used to translate the Pali term *sati*, in the secular context is defined as “the awareness that arises when paying attention on purpose, in the present moment, and non-judgmentally” (26). This definition has three elements to characterize mindfulness as: (i) our innate ability of attention to bring our experiences to the forefront of our awareness; (ii) a process of doing so with an intention of directing it toward the present-moment experiences, without judging them as likable or dislikeable, pleasant or unpleasant, “good” or “bad”; and (iii) awareness with certain qualities that arises as a “result” of applying the ability and the process, which include openness, receptivity, spaciousness, and “stillness” or steadiness that is able to hold any “movement” within it, such as thoughts, emotions, body sensations, or any external stimuli coming from our senses.

It is important to note that the scope of what is denoted by the term *mindfulness* in the secular context exceeds the use of mindfulness as a translation of *sati* in the context of Buddhist meditation praxis methods [for an extensive and comprehensive discussion of the differences, please see the special issue on Mindfulness in Contemporary Buddhism, 2011]. Briefly, this mainly stems from a different approach to meditation practice within different traditions of Buddhism, from which mindfulness as a concept made its way into the secular clinical and scientific context. We do not aim to solve this debate here, but merely point out that when we use the term *mindfulness* in this viewpoint article and the related secular approach to mindfulness practice, we mean it in the way in which Jon Kabat-Zinn has originally intended it (27). The term *mindfulness* in secular usage denotes a far broader range of concepts than used in Buddhist philosophy, psychology and praxis (27), including mindful awareness, which is “captured” by different concepts and referent terms depending on the school of Buddhism. Mindful awareness is referred to as *choiceless awareness* in the context of Mindfulness-Based Stress Reduction (MBSR) (28) and Mindfulness-Based Cognitive Therapy (MBCT) (29). Other terms used for it in the secular context is o*pen presence* (30) or *non-dual mindfulness* (31). These distinctions between traditional Buddhist and secular usage of the term *mindfulness* in terms of the breadth of the definition/conceptual capture are important to note in relation to identifying the mechanisms affording change, both psychological and behavioral. That is, when researching the mechanisms and effects of mindfulness on cognition, emotional regulation and neural dynamics, it is important to be precise regarding the definition and specific aspect(s) of mindfulness (i.e., ability, process or “result”) being studied.

Mindfulness practice as incorporated in Mindfulness-Based Interventions (MBIs) is contrasted with more effortful concentration-based practices, such as taught in Theravada Buddhism (32). The traditions of Buddhism most closely aligned with mindfulness approach intrinsic to MBSR and MBCT are Dzogchen and Mahamudra of Tibetan Buddhism, which take more gentle and effortless approach to practice by letting go of any striving in achieving any particular mental state and simply resting in a present-centered awareness that is non-preferential to the experiential content, free of emotional reactivity to it and conceptual elaboration upon it, whilst being cognizant of experiences as they arise and dissolve in awareness (27, 31). Hence, the attitudes toward mindfulness practice encouraged by MBSR and MBCT include curiosity toward experience, acceptance of what is there to be experienced (which does not entail a passive resignation but rather openness and receptivity as an opposite of experiential avoidance and suppression), non-striving, non-judging one's practice and oneself, and adopting a beginner's mind (suspending preconceived ideas and beliefs about the experiential content and one's identity).

### Mindfulness Skills

The list of beneficial skills afforded by mindfulness practice is potentially long. Here, we will mention a few that we consider to be most relevant to the discussion of the relevance of mindfulness to the context of adopting to challenges presented by the COVID-19 pandemic.

Mindfulness skills captured by the self-report measure *Five Facet Mindfulness Questionnaire* (FFMQ) (33) include: *observing, non-judging, non-reacting, acting with awareness*, and *describing*. *Observing* involves deliberately turning toward and noticing present-moment experiences during daily activities, such as body sensations during walking, showering and eating, or sensory stimuli, such as sounds, smells, or sights. *Non-judging* taps into the tendency to judge one's experiences, emotions, feelings and thoughts, as irrational, inappropriate, or bad, as well as the tendency to be critical of oneself more generally. *Non-reactivity* refers to the ability to be aware of the distressing feelings, thoughts or images without getting caught up in them, noticing them in a decentred way, letting go of them, and returning to feeling calm soon after they have passed. *Acting with awareness* measures the propensity to get distracted from the present-moment experience by mind-wandering (day-dreaming, worrying), running on automatic pilot, and rushing through activities without giving them attention. *Describing* refers to the ability to put one's sensations, feelings, thoughts, beliefs, opinions into words and the tendency to do so. Although there is somewhat of a debate as to whether the ability to act with awareness could be captured by its “opposite”–the propensity for “mindlessness” or lapses of attention, or whether ability to describe is one of the core mindfulness skill [for an in-depth discussion see (34)], there is a consensus that the other three facets, *observing, non-judging*, and *non-reacting*, constitute the core of mindfulness as a trait, both as a personality disposition as well as acquired through formal (e.g., meditation) and informal (daily life) mindfulness practice.

### Transdiagnostic Mechanisms of Action and Implications for the COVID-19 Pandemic

Detailed accounts of the mechanisms underpinning transdiagnostic efficacy of mindfulness practice have been elaborated in relation to depression relapse prevention (35) and more generally for managing psychopathology, as well as promotion of mental health and well-being (36). Wielgosz and colleagues (37) have provided the most recent review of the current understanding of how skills (or capacities) acquired through mindfulness practice translate into its efficaciousness across the psychopathologies, including depression, anxiety, post-traumatic stress, eating disorders and substance abuse by mapping them onto cognitive and affective constructs of the Research Domain Criteria matrix adopted by the NIH (38). Here we briefly outline the most established effects and mechanisms of transdiagnostic efficacy before discussing the relevance of mindfulness to COVID-19 context.

Neuroticism has an established link to psychopathology, both as an efficient marker of non-specified general risk for the common mental disorders (CMDs) (39) and through the association with the CMDs (40). Neuroticism is strongly inversely correlated with *non-reacting* and *non-judging* as dispositional mindfulness traits (41). The relationship between mindfulness and psychological well-being is mediated by the self-compassion (42), with self-compassion being the indirect gain (i.e., not explicitly taught) of the MBCT (43). Self-compassion attenuates anxiety after an ego-threat, and an increase of self-compassion over a one-month period has been shown to augment psychological well-being (42). The practice of mindfulness thus promotes psychological well-being directly via decreased neuroticism due to the acquisition of *non-judging* and *non-reacting* skills, with the benefits being further enhanced by the increases in self-compassion. Self-compassion in the face of negative thoughts has emerged as a key component of the mechanism of change afforded by MBCT (35). Neuroticism as a trait associated with increased stress vulnerability also links to the direct effect of mindfulness on psychological stress reactivity (43).

The relationship with experiences as transient events in the mind attained via *observing, non-judging*, and *non-reacting* skills is akin to the concept of *decentering* in Cognitive-Behavioral Therapy (CBT); learning to experientially decenter through CBT has been linked to the CBT's efficacy in depression relapse prevention (44). Other terms related to this skill are *cognitive diffusion* (45) and *dereification* (46). The experiential mode captured by these terms stands in contrast with self-referencing (identification or fusion with the thoughts and experiences as “me” or “mine” rather than perceiving them as passing events in the mind), and is distinct from the states of dissociation, depersonalization, or derealisation that are associated with psychopathology. This non-elaborative (i.e., simply noticing/observing without reappraisal), non-judgmental, and non-reactive way of processing and relating to experiences, whether they are thoughts, feelings or body sensations, characterize mindfulness as an emotion regulation strategy (47).

Increased self-referencing has been linked to a number of common mental disorders; for example, in a form of rumination in depression or paranoid thoughts in psychosis and schizophrenia. Self-referencing has been linked to the function of the Default Mode Network (DMN) (48). The DMN hyperactivity and over-connectivity has been observed in schizophrenia and depression (49), predicted the post-traumatic stress disorder (50), and is altered in insomnia disorder (51), amongst other psychopathologies (we mention most relevant to the impact of COVID-19 on mental health). The ability to attenuate self-referential processing associated with the DMN activity is enhanced by mindfulness practice (52). This, in part, appears to underpin brain's increased efficiency of information processing (53), relapse prevention in depression (54), and general well-being associated with mindfulness (36).

As argued by Brewer et al. (55) and demonstrated using functional magnetic resonance imaging neurofeedback in conjunction with subjective reports, the DMN's sustained activity, and particularly that of the posterior cingulate region, when processing self-related content (e.g., sensations, memories, emotions, thoughts) may represent “getting caught up in” one's experiences rather than narrative self-referential processes *per se*. Hence, the effect of mindfulness practice on downregulating the DMN activity associated with “sticky” narrative self-referencing alone theoretically underpins its utility for the prevention and management of CMDs (56), as well as for the promotion of mental health and well-being (38).

Related to this is the development of the capacity to disengage from *attention capture* by future- or past-orienting thinking [self-projection, (57)]. Self-projection, also associated with the function of the DMN, is known to be biased toward negative affect (e.g., regret about the past or worry about the future) and maladaptive thinking patterns more generally (58). The inability to disengage from such patterns leads to repetitive negative thinking or proliferation of ruminative self-referential thought, contributing to depression, anxiety, addictive craving, and general stress reactivity (59–61). *Attention capture* is not limited to internal events (e.g., thoughts); for example, visual stimuli of negative valence create stronger fixation than neutral ones (62). In addition to attenuating the activity of DMN, mindfulness training is thought to reduce *attention capture* and enable more efficient disengagement when the fixation occurs via enhanced conscious executive control and entrainment of automatic regulatory circuits (46), as well as improved interoception (63, 64). Reductions in *attentional capture* is thus another important transdiagnostic mechanism by which mindfulness practice can ameliorate the formation of new, resurfacing of past, and exacerbation of the present mental health issues in the context of COVID-19.

Next, we consider the notion of the *beginner's mind* (*shoshin* in Japanese used in Zen Buddhism) that is not currently well-conceptualized in the cognitive approaches to mindfulness. It refers to having an attitude of openness and readiness to experience even the most mundane and repetitive mental events as new, fresh, and free of preconceptions. We showed attenuated startle habituation, which we conceptualized as an index of openness toward repetitive aversive stimuli, in experienced mindfulness practitioners (65). Buddhist psychology posits that one of the reasons for the discontent or dis-ease that even people with no psychiatric diagnosis often experience is a rapid habituation to sensory stimulation, leading to wanting new or higher intensity experiences. In healthy meditation-naïve adults, faster startle habituation is associated with impulsivity, behavioral disinhibition, and sensation seeking (66). Mindfulness practice might thus allow for fresh and alert attention to each incoming stimulus, no matter its valence or familiarity. This might lead to experiential novelty and appreciation of even the most mundane (one of the functions of a “raisin exercise” that opens the MBSR programme is to do just that—to provide an experience of novelty and appreciation of the sensory experience of touching, smelling, and savoring a raisin by adopting mindful awareness). This effect of mindfulness practice in the context of COVID-19 might serve to reduce aggressive or violent behaviors driven by increased irritability and impulsivity that could be brought about by the strict social distancing measures, such as lockdown or quarantine.

Additional relevance of the *beginner's mind* is its possible link to creativity through the ability to see things in a new light and from “out-of-the box” perspectives. In the words of Shunryu Suzuki who popularized the notion of the *beginner's mind* in the book *Zen Mind, Beginner's Mind* (67): “In the beginner's mind there are many possibilities, in the expert's mind there are few.” [p. 21]. Long-term mindfulness meditators exhibit higher divergent thinking (an aspect of creativity assessed by the performance on Alternative Uses Task), which was found to correlate with their mindfulness practice expertise and to be accompanied by an inverse relationship with inter-hemispheric functional connectivity between the main nodes of the DMN (medial prefrontal and posterior cingulate areas) (68). Previous research has linked divergent thinking with greater ability for creative problem solving, over and above the effects of intelligence or expertise (69). Together, these findings present a possibility that the practice of mindfulness might facilitate creative problem solving when dealing with the social and economic aftermath of the pandemic, on both individual and governmental levels.

The final aspect of COVID-19 pandemic that we consider where mindfulness mechanisms might have an important beneficial implication and application is dealing with grief. Grief is a natural response to loss, which can take many forms in the context of COVID-19 pandemic: the death of a loved one; a loss of job, business or important relationship; deterioration of physical and/or cognitive functioning due to COVID-19 illness; a loss of mental equilibrium; or a loss of motivation, sense of purpose and meaning in the face of persistent uncertainty and existential threat.

The beneficial effects of mindfulness on dealing with loss and grief are commonly assumed, with many free resources having been made available for using mindfulness to deal with grief since the start of the pandemic. The very few empirical studies that have assessed the effect of mindfulness-based interventions on grief have indicated promising effects, yet to be confirmed in more rigorously designed larger-scale studies. MBSR in chronic pain patients (70) was found to facilitate a quicker transition through the initial stages of grieving process as compared with the control group of patients seeking or receiving medical assistance. MBSR in breast cancer patients (71) showed significant improvements in existential well-being as well as the reduction in a number of self-identified losses and associated grief.

Theoretical models based on the known mechanisms of mindfulness have been proposed for the use of mindfulness practices for treatment of traumatic and complicated bereavement [e.g., (72)]. MBCT in bereaved individuals was found to significantly improve both executive function and emotion regulation by alleviating emotional interferences on cognitive functions (73), as well as to reduce self-reported anxiety concurrent with increases in self-reported mindfulness that were associated with inter-network reorganization within the brain during the resting state (74).

The Kübler-Ross grief model (75) postulates five stages that those who go through a grieving process may experience, each associated with a distinct emotion: denial, anger, bargaining, depression, and acceptance. Acceptance is inherent to mindfulness process as an attitude or orientation adopted during mindfulness practice toward the experiential content, supporting non-reactivity and experiential openness (32). Mindfulness practice when dealing with loss and grief can bring about an understanding and acceptance of transient and ever-changing nature of all our experiences, whether mental (thoughts, emotions, body sensations) or physical (events, things, relationships). This experiential understanding of all phenomena as being “impermanent” (76) might prove to be an important mechanism for promoting positive adaptation to a highly unpredictable and constantly changing landscape of COVID-19 pandemic. Mindfulness practice has also been shown to increase the sense of meaning, rather than search for meaning *per se*, in a longitudinal study (77), suggesting that grief due to the loss of purpose and meaning, which might be brought about by rapidly changing circumstances, could be regained through mindfulness practice.

## Discussion

### Mindfulness Provision for COVID-19: Now and the Road Ahead

Given the outlined relevance of mindfulness practice and associated skills to dealing with the mental health crisis as secondary to the COVID-19 pandemic, we now discuss its current place within the mental health advice and provision offered through the National Health Service (NHS) in the UK (with implications for health care services elsewhere), as well as offer suggestions for increasing the benefit that could be afforded by a greater exposure and more coordinated implementation.

In recognition of the psychological effects of social distancing and isolation on the UK population, Public Health England's *Every Mind Matters* platform has launched new advice, focused on looking after people's mental well-being during the coronavirus (COVID-19) pandemic. It includes a tailored COVID-19 Mind Plan with content for dealing with specific mental health and well-being issues such as anxiety, stress, low mood and sleep disturbances by signposting the individuals to activities such as mindful breathing exercises, help reframing unhelpful thoughts, and muscle relaxation. The *NHS in Mind* app, rolled out at the end of March 2020, provides a set of free resources designed to help NHS staff to deal with high anxiety, panic and fatigue whilst dealing with the unprecedented demands of their profession in the context of COVID-19. The resources offered include mindfulness-based approaches, such as a 3-min breathing space. *The Mindfulness Initiative* has complied the list of online resources with the NHS staff in mind that could be accessed via their website.

Whilst we welcome such recommendations and signposting to freely available mindfulness-related resources for the general public and the NHS workers, our opportunistic survey of uptake by the target groups has revealed that the engagement with the free online resources particularly amongst the healthcare professionals is very low, with about 1–2% accessing the recommended materials and <1% doing so consistently. Experienced mindfulness instructors across the UK have been providing free Zoom sessions for the NHS staff during their shifts at different times of day across the weekdays and the weekend to enable flexible participation. However, participation has been limited to single digit numbers of NHS professionals per Zoom group/session. Given how supportive mindfulness practice can be for this target group, the reasons for the low uptake need to be understood. According to the mindfulness instructors we have surveyed, the engagement with the Zoom mindfulness sessions during staff break periods appears to be higher in the NHS trusts/hospitals where it is encouraged by the management.

Our online focus group (*N* = 11, all female, comprised of the general practitioners and nurses working for the National Health Service (NHS) in London, UK) that explored the reasons for the low uptake amongst the healthcare professionals suggest a number of possible reasons. First, these highly altruistic individuals have difficulty in “giving care to themselves” even though they recognize the benefits that mindfulness practice can offer them in general, and at this time in particular. Second, a popularized notion that mindfulness expertise development requires at least 10,000 h of practice creates the erroneous belief that extensive practice is required to start experiencing the benefits of mindfulness practice. Being close to burn-out due to the workload, plus having to balance long shifts with family responsibilities, the idea of starting something that will require many hours of practice before the tangible effects will be felt as off-putting. 10,000 h of practice might be a useful criterion to apply for research purposes to define mindfulness practice expertise in the absence of established and reliable objective markers, but it appears to be unhelpful for encouraging individuals to initiate mindfulness practice. Even a simple instruction for mindful emotion regulation of pain experience in meditation-naïve individuals produced the same phenomenological and neural effects as would be expected in experienced mindfulness practitioners (78). A clear distinction should be made when providing a rationale for mindfulness-based approaches between the dose-related effects of mindfulness practice in terms of skills' transfer from state to trait vs. potential benefits gained from mindfulness as a state induced by a single practice session with expected ‘spill over’ effect, even if short-lived at the early stages of practice development, for dealing with daily pressures and stressors.

Related to this is a common belief that mindfulness practice requires effort. Again, in the context of exhaustion faced particularly by the health professionals, this belief is counter-productive in encouraging this population to test the benefits of mindfulness practice for themselves. Instead, the information on mindfulness resources should emphasize that secular mindfulness approach rests on the notion of non-striving; the only “effort” required is in remembering to come back to the present-moment experience whenever the mind becomes distracted by or caught up in the experiences. Mindfulness should be presented as an opportunity to create space, however brief, for diffusion of daily trauma that the mental health professionals in particular are exposed to.

We would also like to suggest providing more in-depth rational for mindfulness practice in terms of its benefits when offering/recommending it to the target populations to fully harness its potential as a preventative cost-effective public intervention. The signposting materials we have surveyed thus far tend to present it as one of the alternatives to other methods of coping, such as distraction by a pleasurable activity, hobby or similar. However, distraction can be adaptive or maladaptive as an emotion regulation strategy depending on whether it is combined with acceptance or avoidance (79). Acceptance has been shown to be associated with positive psychological outcomes [e.g., (80, 81)]. Emotional avoidance, on the other hand, is associated with higher levels of anxiety and affective distress [e.g., (82)]. Nakamura and Orth (83) proposed the distinction between active acceptance, associated with positive psychological outcomes, and resigning acceptance, associated with negative psychological outcomes, and showed active acceptance to be an adaptive response to unchangeable situations. Active acceptance involves experiential openness (84), which is facilitated by mindfulness practice, and is *one* of the attitudes toward the experiential content adopted during mindfulness practice. Therefore, a combination of mindfulness practice followed by a pleasurable activity and hobby may have a more positive effect on well-being than distraction alone, which is likely to be used as avoidance-type coping with stress and anxiety in COVID-19 context.

We have also found in our own experience of signposting to the freely available online resources as well as offering virtual mindfulness courses and drop-in mindfulness sessions to the NHS staff, University students and University staff (both academic and professional) that there is a great appreciation for the group setting, in terms of support of the contact with a mindfulness instructor for the understanding of the rational and the know-how of mindfulness practice *per se*, as well as the normalizing and supportive effects of shared group experience. Therefore, our recommendation for the provision of mindfulness-based support during the COVID-19 pandemic is to offer free virtual mindfulness sessions/courses with trained and experienced mindfulness instructors whenever possible, in addition to signposting to free online resources that could be supportive for personal practice in between regular virtual group sessions.

One notable “success” story in terms of uptake from our survey of online resources is *Headspace*, a private company teaching meditation via a website or a phone app, which has seen a surge in the uptake of its products since mid-March 2020, most notably a doubling of the downloads of the app; 14-fold increase in users completing a free guided meditation for relieving stress; 110% increase in the usage of a “buddy” support; and 70% increase in free live group meditation starting-out sessions. The latter two figures highlight the need for inter-personal support and the value of shared practice experience sought by the users at this time. In recognition of the benefit that mindfulness practice can bring particularly to the health professionals, *Headspace* has been offering free access to its products for US healthcare professionals working in public health settings and all NHS staff to support them in dealing with the present crisis. Given that *Headspace* has a strong emphasis on establishing the evidence-base for their products through collaborative partnerships with academics and is highly popular at a workplace as well as amongst younger users, utilization of its products might be worth considering by the policy makers in the UK and in other English-speaking countries when developing strategies around coordination of mindfulness-based support for mitigating mental health pandemic secondary to COVID-19. Additionally, international collaborative effort is required to develop multilingual evidence-based free online resources to support populations in the face of deteriorating economic conditions globally, and particularly in low and middle income countries.

The intra- and inter-national collaborations on mental health management, provision, and research are essential in dealing with the challenges of the present pandemic and in preparing the best and timely response for the possible future ones. Xiang et al. (2) called for a joint international collaboration to combat mental health challenges during the global COVID-19 pandemic faced by the mental health professionals due to the lack of relevant guidelines and stretched mental health resources in particular. Holmes and colleagues (85) stressed the importance of high-quality data collection on the mental health effects of the COVID-19 pandemic across the whole population and vulnerable groups.

We would like to propose that in addition to understanding the effects of the pandemic on mental illness, the pandemic presents an unprecedented opportunity for studying the factors for resilience, and particularly the role of mindfulness practice, whether in the form of meditation, yoga, qigong, or other, across countries and cultures. Systematic research is urgently needed to examine if adopting these mindfulness-based approaches can not only offset many of the negative mental health consequences, but also help individuals channel the COVID-19-related stress toward positive growth and resilience (86).

The research should also consider the possibility of “negative effects” of mindfulness. As many other phenomena, mindfulness practice appears to follow a non-monotonic or inverted U-shaped trajectory where positive effects at lower to moderate “doses” might turn negative at higher “doses” (87). The research into the “negative effects” needs to make a clear differentiation, mostly lacking in previous research, between what constitutes “negative effects” *resulting* from vs. difficult/challenging experiences which might arise *during* mindfulness practice. This research also needs to be better formulated conceptually, as currently it is heavily culturally relative/biased in terms of what constitutes a “positive” vs. “negative” effect.

Mindfulness developed through the formal practice extends to all aspects of one's life, resulting in greater enjoyment of hobbies, enhancement of inter-personal interactions, and pro-social engagement. The latter will support social cohesion in the times when cooperation is essential, and potentially reduce engagement in conspiratorial and otherwise antagonizing approaches as a way of coping with the disruption posed by the pandemic to what once was the normal order. Hence, the research into the effects of mindfulness practice upon the individuals should extend to understanding its effects on groups and societies at the time of extreme challenge. Schlosser and Bond (88) have observed positive effects of an intervention incorporating elements of different mindfulness-based approaches on team cohesion, team values, and willingness to support each other in a crew of six astronauts during training in isolation over a two-week period.

## Conclusion

Mindfulness-based approaches appear well-suited to deal with the challenges presented by the time of unpresented uncertainty, change, and loss, which can take many forms in the context of COVID-19 pandemic. Mindfulness practice facilitates acceptance of the uncomfortable, difficult, and painful experiences, allowing them to simply be, feeling them as they are without judgement, being present with them until we are ready to let go, and thereby opening ourselves to new experiential and behavioral possibilities. Mindfulness as a way of being exemplifies an approach to life captured by the American poet Robert Frost: “The best way out is always through.” And it is our thesis that it is.

## Data Availability Statement

The original contributions generated for this study are included in the article/supplementary material, further inquiries can be directed to the corresponding author/s.

## Author Contributions

EA, RP, and VK contributed to the conception of the manuscript and its overall structure. KS contributed to manuscript preparation. EA wrote the first draft of the manuscript and revised the manuscript for the final version. VK, RP, and KS provided feedback and contributed to the revision. All authors contributed to the article and approved the submitted version.

## Conflict of Interest

The authors declare that the reported surveys of the MBSR and MBCT instructors providing online mindfulness sessions to the NHS health workers and the online providers of mindfulness-based content (including Headspace) were conducted in the absence of any commercial or financial relationships that could be construed as a potential conflict of interest. The authors declare that the research was conducted in the absence of any commercial or financial relationships that could be construed as a potential conflict of interest.
